# Diverse CO_2_-Induced Responses in Physiology and Gene Expression among Eukaryotic Phytoplankton

**DOI:** 10.3389/fmicb.2017.02547

**Published:** 2017-12-19

**Authors:** Gwenn M. M. Hennon, María D. Hernández Limón, Sheean T. Haley, Andrew R. Juhl, Sonya T. Dyhrman

**Affiliations:** ^1^Lamont-Doherty Earth Observatory, Biology and Paleo Environment, Palisades, NY, United States; ^2^Department of Earth and Environmental Sciences, Columbia University, New York, NY, United States

**Keywords:** algae, carbon concentrating mechanism (CCM), C4 CCM, biophysical CCM, photorespiration, transcriptomics

## Abstract

With rising atmospheric CO_2_, phytoplankton face shifts in ocean chemistry including increased dissolved CO_2_ and acidification that will likely influence the relative competitive fitness of different phytoplankton taxa. Here we compared the physiological and gene expression responses of six species of phytoplankton including a diatom, a raphidophyte, two haptophytes, and two dinoflagellates to ambient (~400 ppm) and elevated (~800 ppm) CO_2_. Dinoflagellates had significantly slower growth rates and higher, yet variable, chlorophyll *a* per cell under elevated CO_2_. The other phytoplankton tended to have increased growth rates and/or decreased chlorophyll *a* per cell. Carbon and nitrogen partitioning of cells shifted under elevated CO_2_ in some species, indicating potential changes in energy fluxes due to changes in carbon concentrating mechanisms (CCM) or photorespiration. Consistent with these phenotypic changes, gene set enrichment analyses revealed shifts in energy, carbon and nitrogen metabolic pathways, though with limited overlap between species in the genes and pathways involved. Similarly, gene expression responses across species revealed few conserved CO_2_-responsive genes within CCM and photorespiration categories, and a survey of available transcriptomes found high diversity in biophysical CCM and photorespiration expressed gene complements between and within the four phyla represented by these species. The few genes that displayed similar responses to CO_2_ across phyla were from understudied gene families, making them targets for further research to uncover the mechanisms of phytoplankton acclimation to elevated CO_2_. These results underscore that eukaryotic phytoplankton have diverse gene complements and gene expression responses to CO_2_ perturbations and highlight the value of cross-phyla comparisons for identifying gene families that respond to environmental change.

## Introduction

Anthropogenic CO_2_ emissions have increased the concentration of CO_2_ in the atmosphere from ~280 ppm at the start of the Industrial Revolution to ~400 ppm at present (Le Quéré et al., 2012), with levels predicted to rise to ~800 ppm by 2100 (Ciais et al., 2013). CO_2_ in the atmosphere acts as a greenhouse gas, warming the planet, and it dissolves into the oceans decreasing the concentration of carbonate ions and acidifying the water (Feely et al., 2004; Sabine et al., 2004). These changes in carbonate chemistry, called ocean acidification, can increase dissolution of carbonates, favor photosynthesis, and alter other cellular processes (Doney et al., 2009). For example, the differential impact of ocean acidification on phytoplankton growth rates is predicted to have a greater impact on phytoplankton community composition and global primary production than ocean warming and decreases in nutrient fluxes from stratification (Dutkiewicz et al., 2015).

Eukaryotic phytoplankton, in particular, play important roles in aquatic biogeochemical cycles and marine ecosystems. For example, diatoms alone are responsible for ~20% of global primary production (Nelson et al., 1995). Coastal and upwelling ecosystems, where larger eukaryotic phytoplankton thrive, host some of the largest fisheries through efficient trophic transfer (Ryther, 1969). Diatoms and coccolithophores also create biominerals that serve to transport silica (Nelson et al., 1995) and calcium carbonate (Milliman, 1993) with organic carbon from the surface to the deep ocean (Ducklow et al., 2001), sequestering this carbon for potentially thousands of years. In addition, some species of phytoplankton can cause harmful algal blooms (HABs) that disrupt ecosystems and human activities by growing to high concentrations or producing toxins (Trainer and Yoshida, 2014). Dissolved CO_2_ in the oceans is highly variable on regional and seasonal scales (Takahashi et al., 2014) and can be particularly high in coastal upwelling regions (Feely et al., 2008) where eukaryotic phytoplankton are abundant, making it likely that coastal eukaryotic phytoplankton regularly experience large fluctuations in CO_2_. Phytoplankton can change their carbon affinity based on acclimation to different concentrations of CO_2_ (Rost et al., 2003), but this acclimation effect varies by species. Predicting how the future structure of marine ecosystems will change with rising CO_2_ requires an understanding of how shifting carbonate chemistry will impact phytoplankton from diverse eukaryotic lineages (Hallegraeff, 2010; Dutkiewicz et al., 2015).

All phytoplankton share the rate-limiting enzyme of carbon fixation, ribulose-1,5-bisphosphate carboxylase/oxygenase (Rubisco), though the form differs in different groups, e.g., form II in most dinoflagellates (Morse et al., 1995) and form 1D for other red algal lineages (Tabita et al., 2008). Rubisco originated under much higher ambient CO_2_ and low oxygen levels and evolved in eukaryotic phytoplankton lineages as oxygen increased and CO_2_ levels decreased (Tortell, 2000; Young et al., 2012). Rubisco remains vulnerable to fixing oxygen in the place of CO_2_, resulting in the loss of fixed carbon—a metabolic pathway known as photorespiration (Bauwe et al., 2010). Although photorespiration is typically considered to be detrimental to photoautotrophs (Bauwe et al., 2010), it can also serve as a mechanism for phytoplankton to eliminate excess energy in times of high light or variable nutrient environments (Parker and Armbrust, 2005; Bagby and Chisholm, 2015). Rubisco form 1D and II differ in structural (Tabita et al., 2008) and kinetic properties (Badger et al., 1998); form II Rubisco has fewer subunits and a relatively higher rate of oxygen fixation compared with form 1D Rubisco.

Nearly all marine phytoplankton have carbon concentrating mechanisms (CCM) to increase the concentration of carbon around the active site of Rubisco by ~3 to 80-fold in order to favor carbon fixation and prevent oxygen fixation (Badger et al., 1998). The genes involved in CCMs remain largely uncharacterized for most species. Evidence for fixation of carbon into a four-carbon compound (C4 metabolism) has been described for a few species of diatoms (Reinfelder et al., 2000; McGinn and Morel, 2007) and for a coccolithophore (Tsuji et al., 2012). Yet the evidence remains mixed as to whether C4 compounds can be used as a CCM in phytoplankton (Kroth et al., 2008) and whether C4 enzymes are regulated by CO_2_ (Roberts et al., 2007; Kustka et al., 2014). Biophysical CCMs consisting of, at minimum, a bicarbonate pump and a carbonic anhydrase are thought to be potentially more efficient than C4 CCMs (Hopkinson et al., 2011), yet the genes that could be used for biophysical CCMs are multi-copy and diverse even within a single species (Tachibana et al., 2011), making a systematic study across phytoplankton lineages challenging. Large uncertainties persist in the range of potential CCM genes in phytoplankton and what trade-offs might result from regulating CCMs in response to rising CO_2_. Decreased expression of biophysical (Hennon et al., 2015) and C4 CCM gene expression (Kustka et al., 2014) has been observed in the diatom, *Thalassiosira pseudonana*, in response to elevated CO_2_. Concurrent rearrangements in metabolic pathways and physiology have also been observed in *T. pseudonana* (Hennon et al., 2014, 2015), likely as a result of metabolic rearrangement with a decreased need for the energy-consuming CCM. Similar responses for a few well-studied coccolithophores (Rokitta et al., 2012) have been reported. It is an open question how many other ecologically-important species of phytoplankton will alter their CCM and photorespiration genes in response to CO_2_ and what impact this will have on core metabolic processes and growth rate responses.

To examine how CCM and photorespiration genes are modulated in response to CO_2_ and what impact this has on core metabolic processes and growth rate responses, six eukaryotic phytoplankton species from four phyla were cultured under elevated (~800 ppm) and ambient (~400 ppm) CO_2_. These phytoplankton included: a cosmopolitan diatom (*Chaetoceros affinis*), a raphidophyte (*Heterosigma akashiwo*), a calcifying haptophyte (*Gephyrocapsa oceanica*), a non-calcifying haptophyte (*Chrysochromulina polylepis*), and two dinoflagellates (*Alexandrium monilatum* and *Prorocentrum minimum*). All species are ecologically important marine phytoplankton, with four documented to cause HABs: *A. monilatum* (Juhl, 2005; Anderson et al., 2012), *P. minimum* (Heil et al., 2005), *H. akashiwo* (Honjo, 1992), and *C. polylepis* (Dahl et al., 1989). Changes in physiology and gene expression in response to elevated CO_2_ were evaluated for all six phytoplankton to determine the impact of future ocean chemistry on these phyla. Additionally, a comprehensive analysis of all consensus sequences from the same four phyla within the marine microbial eukaryote transcriptome project (MMETSP) database was conducted to assess the potential for these phytoplankton lineages to acclimate to rising CO_2_ based on the expressed gene complement of CCM and photorespiration pathways.

## Methods

### Culturing conditions

Experiments were performed on six species of phytoplankton: *A. monilatum* (CCMP3105), *C. affinis* (CCMP159), *C. polylepis* (CCMP1757), *G. oceanica* (RCC1303), *H. akashiwo* (CCMP2393), and *P. minimum* (CCMP1329), available from the National Center for Marine Algae and Microbiota (NCMA, formerly CCMP) and Roscoff Culture Collections (RCC). All cultures were uni-algal and uni-eukaryotic, but not axenic. Strains were cultured in L1 media (with silica for the diatom *C. affinis*), except *G. oceanica* which is native to open ocean habitats and was grown in a reduced nutrient medium (L1/25 vitamins and trace metals with L1/10 nitrate and L1/15 phosphate), to ensure calcification. The natural seawater for the media base for all species was collected from Vineyard Sound, MA (salinity 32). Cultures were grown at 18°C, except for species with different temperature optima: *A. monilatum* (24°C) and *C. polylepis* (15°C). Light levels in all experimental treatments were ~100 μmol photon m^−2^ s^−1^ with a 14:10 light:dark cycle. Culture conditions are summarized in Supplementary Table 1.

Strains were acclimated to experimental conditions in 100-mL culture flasks for 3 days (or ~1–3 generations). Triplicate experimental batch cultures were inoculated with 25 mL of acclimated culture. Experimental batch cultures were grown in 1-L volumes in 2.5-L polycarbonate bottles under gentle rotation (75 rpm). To control the carbonate chemistry of the water, the headspace of each bottle was purged continuously with either ambient outside air of 400 ppm (±15 ppm) checked with a LI-7200RS CO_2_ Analyzer (LI-COR, Lincoln NB, USA) or a custom gas mix (TechAir, NY, USA) of 800 ppm (± 12 ppm) carbon dioxide with 21% oxygen and balance nitrogen. The gas streams were pre-filtered through 0.2-μm HEPA filters and directed through a sterile glass pipette to break the boundary layer of the media without contacting the surface. The bottles were not bubbled to avoid the adverse effects of turbulence on phytoplankton growth (Thomas and Gibson, 1990; Juhl and Latz, 2002). Cell concentration and carbonate chemistry were monitored over the course of the experiments, which were typically 4–5 days. At this point, experimental batch cultures were harvested for physiological and gene expression measurements. This approach both constrained the biomass to ecologically-relevant levels and permitted the calculation of growth rates with confidence.

### Carbonate chemistry

Differences in media carbonate chemistry were confirmed by measuring pH using an electrode (300728.1, Denver Instruments) calibrated with NIST buffers (pH 4–10). All cultures were grown in media from the same base seawater from Vineyard Sound, Massachusetts with salinity (S) of 32. The total alkalinity (TA) was estimated with an empirical formula derived from an offshore transect from Woods Hole, MA to the mid-Atlantic Bight (TA = 73.4 × S −188.7) (Cai et al., 2010). Calculations of carbonate chemistry parameters were performed by CO2SYS (Lewis and Wallace, 1998) and summarized in Supplementary Table 1. Although all species were grown at ecologically-relevant cell concentrations with headspace continuously purged with elevated (~800 ppm) or ambient (~400 ppm) CO_2_ (Supplementary Table 1), aqueous CO_2_ was drawn down by the phytoplankton as they fixed carbon, resulting in lower mean dissolved CO_2_ concentrations compared to the headspace for some species. Thus, the changes in dissolved CO_2_ concentrations varied between species (Supplementary Table 1). Despite this variability, dissolved CO_2_ concentrations exceeded 400 ppm in all flasks purged with elevated CO_2_ and the dissolved CO_2_ levels in ambient and elevated treatments for each species were offset from each other, with the difference ranging 0.2–0.5 pH units and equating to a difference of ~200–600 ppm dissolved CO_2_ (Supplementary Table 1). For clarity of the manuscript, the treatments are referred to as elevated (~800 ppm) or ambient (~400 ppm) CO_2_ throughout the text.

### Physiological measurements

Daily, 1 mL of each triplicate culture was preserved in 2% Lugols solution (final concentration) for microscopic cell counts. Growth rate within each flask was calculated as the slope of the natural log of biomass (cell abundance) vs. time. There was no evidence of a lag phase in any of the six species, suggesting cultures were appropriately acclimated (Berge et al., 2010), but growth rate was conservatively calculated from only the last 3–5 days of the experiment to most closely represent growth rate at harvest. For harvest, volumes of 10, 25, and 50 mL of each culture were gently filtered on 25-mm, pre-combusted glass fiber filters (Whatman, GF/F) and frozen at −20°C until processing for chlorophyll *a*, total particulate carbohydrates and elemental analyses respectively. Chlorophyll *a* concentration was determined according to Strickland and Parsons (1972). Briefly, the samples were extracted in 10 mL of 90% acetone, vortexed for 15 s, and stored in the dark at −20°C for 12 h. The fluorescence in the supernatant was then measured on a Turner Designs Aquafluor fluorometer before and after acidification with 0.1 N hydrochloric acid. The fluorometer was calibrated with chlorophyll *a* from *Anacystis nidulans* (Sigma). Total particulate carbohydrates (pCHO) were measured according to a modified phenol-sulfuric acid method (DuBois et al., 1956). Filters were extracted in 5% phenol and concentrated sulfuric acid in 15-mL polypropylene tubes at 30°C for 12 h. After removal of the filter, light absorption of the sample at 490 nm was measured using a Hach DR2700 spectrophotometer and compared to a glucose calibration curve. Elemental analysis of particulate organic carbon and nitrogen (POC:PON) was performed by the Nutrient Analytical Services Laboratory at the Chesapeake Bay Laboratory (University of Maryland, Solomon, MD) with a CE-440 Elemental Analyzer following the methods of USEPA (1997). All physiological measurements were normalized to cell concentration from their respective flask.

### RNA extraction and sequencing

At the time of harvest, triplicate 150-mL subsamples of each replicate in each treatment were filtered onto polycarbonate filters (47 mm, 0.2–3 μm depending on species). Filters were immediately flash frozen and stored in liquid nitrogen until extraction. RNA extractions were performed with RNeasy Mini Kit (Qiagen, Valencia, CA), with a slight modification to the lysis procedure. Lysis was performed by adding 1.4 mL of Buffer RLT and ~250 μL zirconium/silica beads (0.5 mm) and vortexing for 1 min at 250 rpm. DNA was removed using TurboDNase (Ambion, Austin, TX). Total RNA extracts of the triplicate cultures for each treatment were pooled for sequencing, quantified using a Qubit fluorometer (Invitrogen, Carlsbad, CA) and verified to be high quality using a 2100 Bioanalyzer (Agilent, Santa Clara, CA). The sequencing libraries were prepared using the Illumina TruSeq RNA Sample Preparation Kit to select mRNA with polyA tails. Library preparation and sequencing of ~50-bp, paired-end reads from each library was performed on an Illumina HiSeq 2000 at the National Center for Genome Resources (Sante Fe, NM).

### Sequence assembly and alignment

Combined assemblies and alignments for sequences from each phytoplankton strain were generated as part of the Marine Microbial Eukaryote Transcriptome Project (MMETSP) described in detail in Keeling et al. (2014). Briefly, sequence reads were pre-processed using SGA (Simpson and Durbin, 2012). After trimming, reads less than 25 bp in length were discarded. Quality-controlled sequences were assembled into contigs with ABySS (Simpson et al., 2009) for each sample. The resulting contigs from all samples from a single strain were combined into one consensus assembly using a combination of assembly tools: CD-HIT-EST (Li and Godzik, 2006), ABySS, CAP3 (Huang and Madan, 1999), GapCloser (SOAP, Li et al., 2008), with redundant contigs removed at a threshold of 98% identity. Reads were aligned to combined assemblies with BWA (Li and Durbin, 2009). Combined assemblies and alignment counts are available from iMicrobe (ftp://ftp.imicrobe.us/camera/combined_assemblies/) or raw reads from BioProject (PRJNA248394) for IDs: *A. monilatum* (control, MMETSP0093; elevated CO_2_ MMETSP0097), *C. affinis* (control, MMETSP0088; elevated CO_2_, MMETSP0092), *C. polylepis* (control, MMETSP0143; elevated CO_2_, MMETSP0147), *G. oceanica* (control, MMETSP1363; elevated CO_2_, MMETSP1364), *H. akashiwo* (control, MMETSP0292; elevated CO_2_, MMETSP0296), *P. minimum* (control, MMETSP0053; elevated CO_2_, MMETSP0057).

### Read count normalization and annotation

Contig counts resulting from sequence assembly and alignment were normalized using the trimmed mean of M-values (function tmm, NOISeq package, R) and then normalized to read counts from ambient CO_2_ controls. Translated contigs (peptide sequences) were annotated with GhostKOALA (Kanehisa et al., 2016) using the KEGG database of genus-level prokaryotic and family-level eukaryotic sequences.

### Orthologous group clustering and comparison

Consensus contigs from the six eukaryotic phytoplankton species were clustered into orthologous groups by OrthoFinder (Emms and Kelly, 2015), and orthologous groups represented in at least two species were kept for further analysis. Orthologous groups that had a greater than 2-fold change (|log_2_FC| > 1) under elevated compared to ambient CO_2_ and total of 10 reads or greater across both treatments (>−16 log_2_ counts per million) were then compared across the six species. Overlaps in CO_2_-responsive orthologous groups were visualized with UpSet (http://caleydo.org/tools/upset/).

### CCM and photorespiration gene identification

Putative CCM genes, biophysical and C4, and photorespiration pathway genes were compiled from the literature. Hidden Markov Model (HMM) seed files were created for each gene using peptide sequences from complete genomes of at least four species belonging to at least three phyla. Seed file genes, applicable citations (Branson et al., 2004; Jitrapakdee et al., 2008; Kroth et al., 2008; Milenkovic et al., 2008; Hackenberg et al., 2011; Tachibana et al., 2011; Tsuji et al., 2012; Nakajima et al., 2013; Romero et al., 2013; Chi et al., 2014; Hennon et al., 2015), and their accession IDs from GenBank are detailed in Supplementary Table 2. Multiple alignments of HMM seed files were performed by MAFFT (Katoh and Standley, 2013) (on setting auto) and checked with Jalview (Waterhouse et al., 2009) (version 2) visualization software. HMM searches for each seed file were performed on translated contigs using HMMER (Eddy, 2011) (version 3.1b2, HMMbuild, HMMsearch) with an E-value cut-off of 10^−4^, the results are summarized in Supplementary Table 3 and contig IDs for each HMM hit are listed in Supplementary Table 4. Putative CCM and photorespiration genes were identified from the six eukaryotic phytoplankton species as well as all available consensus contigs from the same four phyla in the MMETSP database of combined assemblies (ftp://ftp.imicrobe.us/camera/combined_assemblies/).

### Statistical analysis

Differences in physiological measurements between ambient and elevated CO_2_ for each species were tested with student's *t*-test (*t*-test, stats package, R). Due to the pooling of biological replicates in each of the six eukaryotic phytoplankton transcriptomes the significance of differential expression for individual genes was not calculated. Rather, a cut-off of greater than 2-fold change (|log_2_FC| > 1), thought to represent a substantial biological response (Mock et al., 2008; Shrestha et al., 2012), and a minimum of 10 reads or greater across both treatments (>−16 log_2_ counts per million) was used to define contigs with increased and decreased expression under elevated CO_2_. Rather than comparing expression for individual genes, analyses focused on whether changes in sets of genes were statistically significant between treatments. Gene set enrichment, the fraction of pathway genes present in increased or decreased contigs compared to total contigs, was tested for 10 sets defined by KEGG pathways involved in energy, carbon and nitrogen fluxes: photosynthesis (00195), photosynthesis antenna proteins (00196), porphyrin and chlorophyll metabolism (00860), carbon fixation in photosynthetic eukaryotes (00710), tricarboxylic acid cycle (00020), glycolysis and gluconeogenesis (00010), nitrogen metabolism (00910), fatty acid elongation (00062), fatty acid degradation (00071), and biosynthesis of unsaturated fatty acids (01040). The hypergeometric test (phyper, stats, R) was used to test for statistical significance. To test whether the CCM and photorespiration gene sets were significantly differently distributed compared to all genes, two-sample Kolmogorov-Smirnov tests (ks_2samp, SciPy library, Python) were used. Custom python scripts are available on github (https://github.com/MDHDZ91).

## Results and discussion

### Section 1: CO_2_ physiology of six eukaryotic phytoplankton species

**Figure 1:**
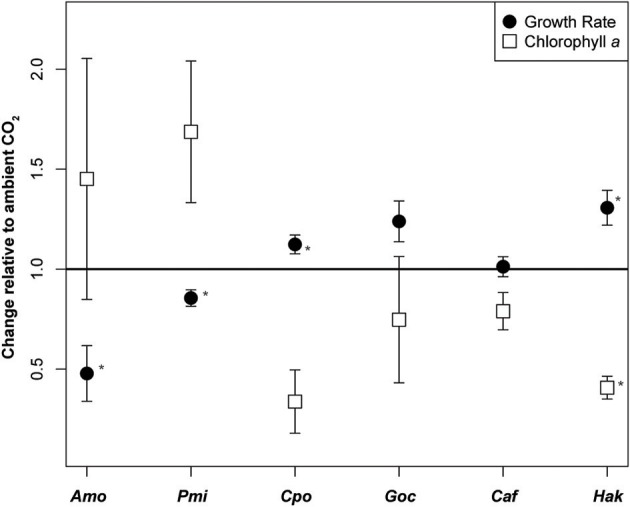
Growth rate response and change in chlorophyll *a* content under elevated CO_2_. Change in growth rate (d^−1^) and chlorophyll *a* (pg cell^−1^) under elevated relative to ambient CO_2_ in six species of phytoplankton. Black circles indicate growth rate response and squares indicate the change in chlorophyll *a* content per cell (mean ± SE). An asterisk represents statistically significant differences in elevated vs. ambient CO_2_ (*p* < 0.05, two-sided *t*-test, *n* = 3). Amo, *Alexandrium monilatum*; Pmi, *Prorocentrum minimum*; Cpo, *Chrysochromulina polylepis*; Goc, *Gephyrocapsa oceanica;* Caf, *Chaetoceros affinis*; Hak, *Heterosigma akashiwo*.

Six eukaryotic phytoplankton species were grown in nutrient replete media under headspaces purged with elevated (~800 ppm) or ambient (~400 ppm) CO_2_ (Supplementary Table 1). Changes in growth rate and chlorophyll *a* per cell showed high variability between species (Table 1). The dinoflagellates, *A. monilatum*, and *P. minimum*, both grew significantly slower in elevated CO_2_ (Table 1), averaging 0.67 ± 0.07 (mean ± 1SE) of the growth rate at ambient CO_2_ (Figure 1). The chlorophyll *a* content trended higher under elevated CO_2_ in both dinoflagellates, although it was highly variable within biological replicates and not statistically significantly different between CO_2_ treatments (Figure 1, Table 1). In contrast, *C. polylepis* and *H. akashiwo* had statistically significant increases in growth rate (Table 1) consistent with previous studies of large diatoms (Wu et al., 2014). Only one species, *H. akashiwo*, had significantly less chlorophyll *a* per cell (Table 1). The differences in the impact of CO_2_ on growth and chlorophyll content in these species may be driven by differences in cell size, Rubisco form or gene complement, disentangling the mechanisms driving physiological differences will be important to accurately predict the future competitive fitness of different groups of eukaryotic phytoplankton.

**Table 1:** Physiology of six eukaryotic phytoplankton grown under ambient and elevated CO_2_.

**Species**	**CO_2_**	**Growth rate (d**^**−1**^**)**		**Chlorophyll** ***a*** **(pg cell**^**−1**^**)**		**PON (pmol cell**^**−1**^**)**		**POC (pmol cell**^**−1**^**)**		**pCHO (pmol cell**^**−1**^**)**	
*Alexandrium monilatum*	Ambient	0.26 ± 0.03		48.67 ± 32.62		175.26 ± 6.92		781.71 ± 62.21		401.71 ± 123.72	
	Elevated	0.12 ± 0.06	*0.02*	70.62 ± 18.39	*ns*	198.69 ± 31.15	*ns*	922.71 ± 145.05	*ns*	571.13 ± 54.47	*ns*
*Prorocentrum minimum*	Ambient	0.65 ± 0.03		1.26 ± 0.38		3.25 ± 1.00		34.24 ± 5.29		28.42 ± 15.71	
	Elevated	0.55 ± 0.04	*0.03*	2.12 ± 0.42	*ns*	5.44 ± 0.23	*0.02*	58.54 ± 5.00	*< 0.01*	43.69 ± 5.76	*ns*
*Chrysochromulina polylepis*	Ambient	0.33 ± 0.02		6.54 ± 5.12		5.51 ± 3.04		45.11 ± 28.87		24.32 ± 5.10	
	Elevated	0.37 ± 0.02	*0.05*	2.21 ± 0.48	*ns*	2.80 ± 0.69	*ns*	15.29 ± 6.52	*ns*	20.84 ± 0.61	*ns*
*Gephyrocapsa oceanica*	Ambient	0.84 ± 0.10		0.10 ± 0.07		0.07 ± 0.04		0.64 ± 0.39		0.17 ± 0.13	
	Elevated	1.04 ± 0.08	*ns*	0.08 ± 0.01	*ns*	0.06 ± 0.01	*ns*	0.54 ± 0.12	*ns*	0.10 ± 0.02	*ns*
*Chaetoceros affinis*	Ambient	0.97 ± 0.02		3.07 ± 0.31		2.06 ± 0.12		14.08 ± 0.75		5.64 ± 2.80	
	Elevated	0.98 ± 0.08	*ns*	2.42 ± 0.43	*ns*	3.46 ± 0.12	*< 0.01*	13.08 ± 2.37	*ns*	6.41 ± 0.45	*ns*
*Heterosigma akashiwo*	Ambient	0.47 ± 0.05		6.37 ± 1.01		3.86 ± 0.64		24.83 ± 4.69		5.54 ± 0.34	
	Elevated	0.61 ± 0.01	*0.01*	2.59 ± 0.48	*< 0.01*	4.94 ± 1.01	*ns*	27.18 ± 4.19	*ns*	24.00 ± 7.72	*0.01*

*Mean ± SD (n = 3) of exponential growth rate (d^−1^), chlorophyll a content (pg cell^−1^), particulate organic nitrogen (PON), particulate organic carbon (POC), and particulate carbohydrate (pCHO) content per cell (pmol cell^−1^). The p-values resulting from a two-sided t-test (n = 3) are indicated in italics where statistically significant, (ns) indicates not significantly different*.

**Figure 2:**
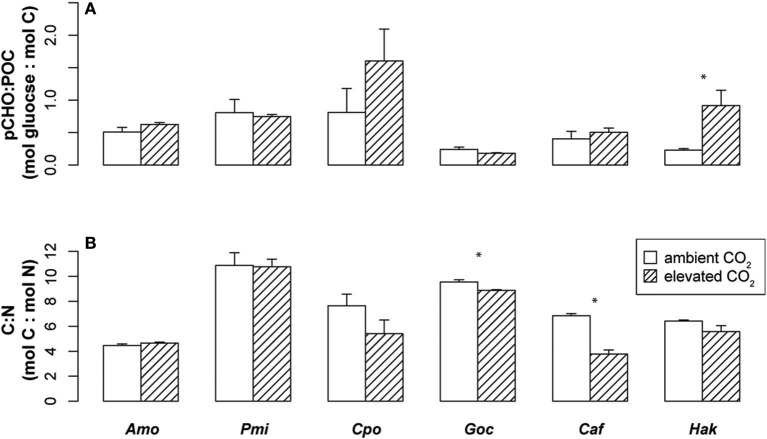
Carbon and nitrogen composition of phytoplankton under ambient and elevated CO_2_. Mean ± SE of **(A)** the ratio of carbohydrate (pCHO units of pmol glucose) to total particulate organic carbon (POC units of pmol carbon) and **(B)** carbon to nitrogen (C:N) for six species of phytoplankton. Asterisks indicate significant differences in means between ambient and elevated CO_2_ (*p* < 0.05, *n* = 3, two-sided *t-*test). Abbreviations are as in Figure 1.

The carbon and nitrogen composition of these six phytoplankton species was also examined for changes under elevated CO_2_ (Figure 2, Table 1). *P. minimum* was the only species that showed a significant change in POC per cell, while the raphidophyte, *H. akashiwo*, was the only species showing significant differences in pCHO per cell and in pCHO:POC. The dinoflagellates did not significantly alter their C:N ratios, while C:N of phytoplankton from other lineages dropped with elevated CO_2_, although this change was only significant in the diatom, *C. affinis*, and the coccolithophore, *G. oceanica* (Figure 2). For *C. affinis*, the shift in C:N was driven by a significant increase in nitrogen cell quota (Table 1), suggesting an increase in nitrate assimilation rate. These results differ from previous studies of phytoplankton communities in mesocosms (Riebesell et al., 2007), yet are similar to the findings of Eberlein et al. (2016) for dinoflagellates suggesting that there may be a wide range of species-specific responses in C:N ratio to elevated CO_2_. It is important to understand changes in phytoplankton elemental composition for predicting future biogeochemistry and marine food web dynamics, as a decrease in C:N ratio could decrease the efficiency of carbon sequestration (Riebesell et al., 2007) while improving quality of phytoplankton as food for higher trophic levels (Elser et al., 2000).

### Section 2: CO_2_-induced changes in gene expression

**Figure 3:**
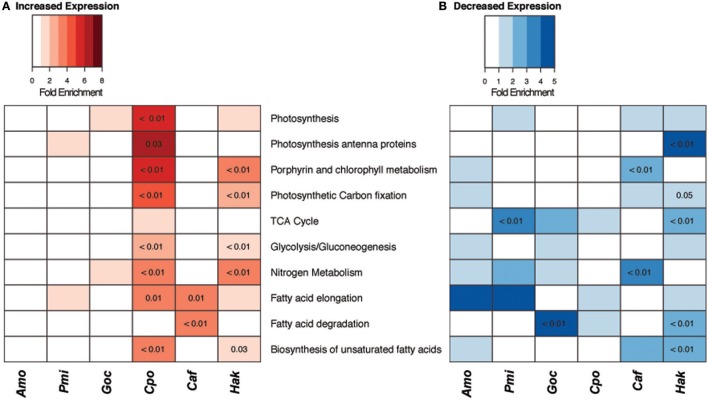
Changes in carbon and nitrogen metabolism gene expression under elevated CO_2_. Gene set enrichment of carbon and nitrogen KEGG pathways with **(A)** increased and **(B)** decreased expression (|log_2_FC| > 1, logCPM > −16) for six species of phytoplankton. Fold enrichment of each category is indicated by color intensity, numbers indicate *p*-values for gene sets with significant enrichment (*p* < 0.05, hypergeometric test).

Transcriptomes were sequenced for each species to investigate how these phytoplankton shift metabolic pathways to acclimate to elevated CO_2_. The changes in core carbon and nitrogen metabolic pathways differed among the six species (Figure 3). *C. polylepis* and *H. akashiwo* were the two species with the highest number of gene sets with significantly increased expression, including many carbon and nitrogen metabolism genes, particularly biosynthesis of unsaturated fatty acids (Figure 3A). The increased expression of these biosynthesis genes in *C. polylepis* and *H. akashiwo*, which also had significantly higher growth rates under elevated CO_2_, likely corresponded to faster rates of lipid biosynthesis. Both *C. polylepis* and *H. akashiwo* also had significant enrichment of Glycolysis/Gluconeogenesis pathway genes without a corresponding significant enrichment of TCA pathway genes (Figure 3A), suggesting that the genetic underpinning for accumulation of carbohydrates in these two species (Figure 2A) may stem from favoring gluconeogenesis over glycolysis and respiration. The diatom, *C. affinis*, had significantly enriched expression of fatty acid elongation as well as degradation genes (Figure 3A) with no significant change in growth rate (Table 1), suggesting there may be futile cycling of lipid synthesis and degradation. *C. affinis* also displayed decreased expression of nitrogen and chlorophyll metabolism genes with significant gene set enrichment (Figure 3B), corresponding to the significant shift in C:N ratio and lower chlorophyll *a* content (Table 1). *G. oceanica* presented a more muted gene expression response compared to other form 1D Rubisco species with fewer significant changes in gene set enrichment including a decrease in fatty acid degradation pathway gene expression (Figure 3B). This muted metabolic gene expression response corresponded with a relatively minor growth rate response for *G. oceanica* to elevated CO_2_ (Table 1).

The dinoflagellates, *A. monilatum*, and *P. minimum*, also displayed few significant changes in carbon and nitrogen pathway gene expression (Figure 3). The exception to this being the reduced expression of TCA cycle genes by *P. minimum*, which may help to explain the decrease in growth rate (Figure 1). A limited transcriptional response of the dinoflagellates has been observed in experiments with nutrient limitation (Harke et al., 2017) and elevated temperatures (Barshis et al., 2014). The limited transcriptional response to environmental perturbations may be largely explained by the underlying regulatory mechanisms of cell physiology occurring at the translational or post-translational level for an estimated ~73–90% of dinoflagellate genes (Lin, 2011; Murray et al., 2016). Regardless, the limited change in carbon and nitrogen pathway genes for this group is consistent with their lack of significant change in carbon and nitrogen partitioning (Figure 2).

**Figure 4:**
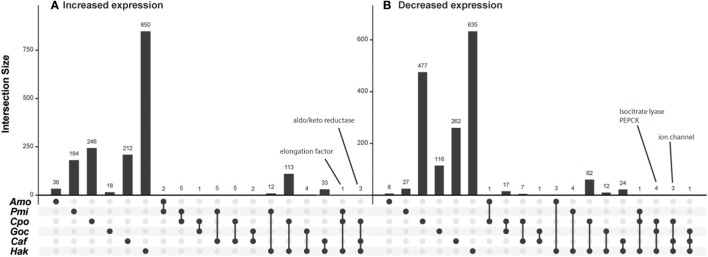
Intersection of orthologous genes with shared expression patterns under elevated CO_2_ for phytoplankton species. Intersection size (number of shared orthologous genes) is indicated by bars for each grouping of species indicated by circles below the axis. Intersections of genes with **(A)** increased expression (log_2_ Fold Change > 1) and **(B)** decreased expression (log_2_ Fold Change < −1). Intersections with no shared orthologous genes are omitted, species abbreviations are as in Figure 1.

To investigate whether there were any genes not classified into KEGG pathways that responded similarly to CO_2_ across species, the genes were also grouped into orthologous clusters. Despite similarities in physiological responses between species (Table 1), there was almost no shared expression change in orthologous genes (Figure 4). No orthologs showed an expression pattern that was shared by more than three of the six species examined, and those that did share expression patterns among three species were frequently hypothetical proteins with unidentified function or genes with general functions such as transcription (e.g., elongation factor) and oxidoreductase activity (e.g., aldo/keto reductase) (Figure 4). A few of the genes that had decreased expression across three species (Figure 4B) have putative roles in photorespiration (isocitrate lyase, ICL), C4 metabolism (phosphoenolpyruvate carboxykinase, PEPCK), and ion balance (ion channel). Decreased expression of putative CCM and photorespiration genes with elevated CO_2_ agrees with previous work with diatoms (Kustka et al., 2014; Hennon et al., 2015). Nevertheless, the analysis of metabolic gene sets and orthologous groups suggest limited commonality in the transcriptional response of these six species to elevated CO_2_.

**Figure 5:**
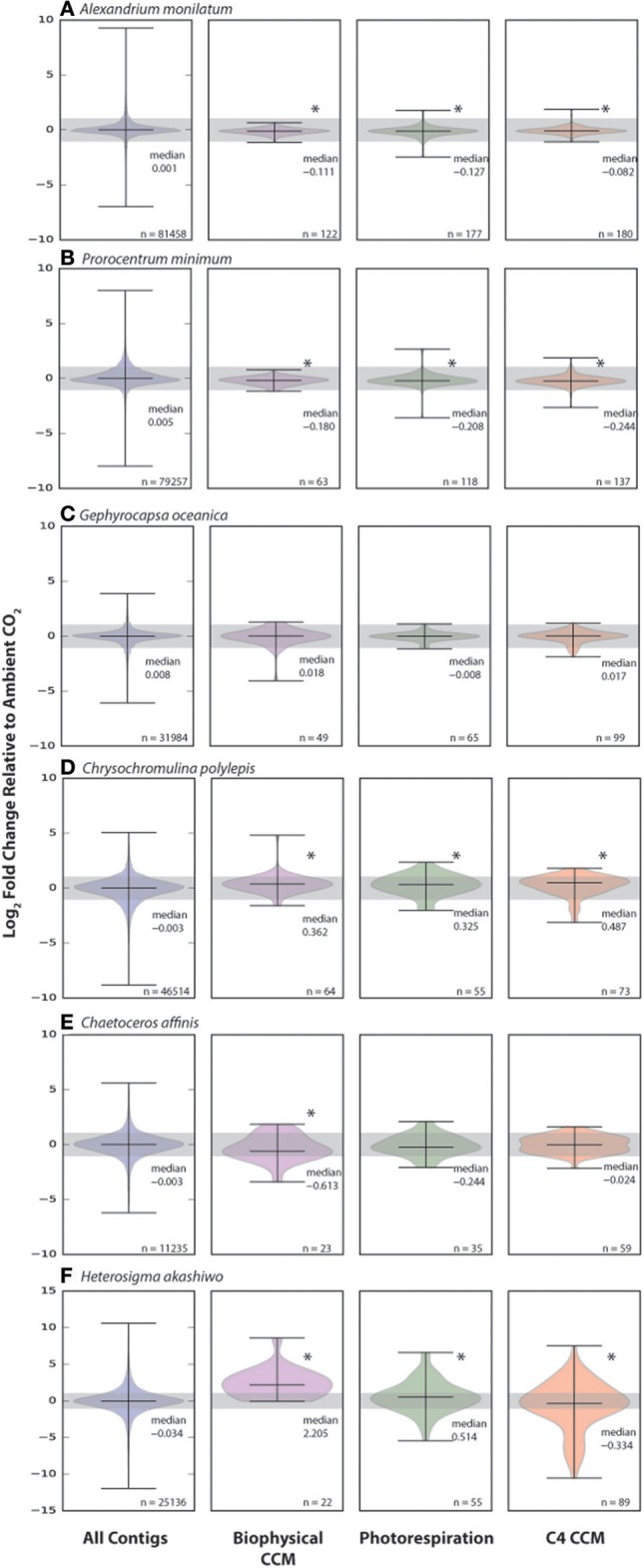
Change in distribution of carbon concentrating mechanism (CCM) and photorespiration genes in six eukaryotic phytoplankton under elevated CO_2._ Distributions of the expression (log_2_ Fold Change) at elevated CO_2_ relative to ambient CO_2_ of all expressed contigs, biophysical CCM, photorespiration pathway genes and C4 CCM genes for *Alexandrium monilatum* **(A)**, *Prorocentrum minimum* **(B)**, *Gephyrocapsa oceanica* **(C)**, *Chrysochromulina polylepis* **(D)**, *Chaetoceros affinis* **(E)**, *Heterosigma akashiwo* **(F)**. Asterisks indicate significant difference in expression distribution from that of all expressed contigs (Kolmogorov-Smirnov test, *p* < 0.05). Distribution median and number of genes per distribution (*n*) given on the figure.

To more comprehensively explore the possible CO_2_-related shifts in CCM and photorespiration genes, Hidden Markov Model (HMM) searches were performed to identify genes that match the patterns of known homologs (Supplementary Table 2). The expression patterns of CCM and photorespiration homologs were compared to the distribution of all genes (Figure 5). Similar to the other gene expression and physiology measures, there were a large variety of responses between species. The two dinoflagellates, *A. monilatum*, and *P. minimum*, had significantly different distributions of CCM and photorespiration gene expression compared to all expressed genes (K-S test, *p* < 0.05, Figures 5A,B). However, few genes in the dinoflagellates had fold changes greater than the 2-fold change cut-off to be canonically considered biologically significant (Table 2). The median expression of CCM and photorespiration genes declined by 6–8% in *A. monilatum* and 11–16% in *P. minimum*, agreeing with the expectation that elevated CO_2_ would result in a decrease in the use of these pathways and with previous studies showing a decrease in carbonic anhydrase expression in other dinoflagellates (Van de Waal et al., 2013, 2014). These data suggest that CCM and photorespiration genes may be responding to elevated CO_2_. However, the growth rate effects (Table 1, Figure 1) are opposite of expectations for a decreased CCM, leaving the question open as to why dinoflagellates in this study grew more slowly under elevated CO_2_. One possibility is that *P. minimum* and *A. monilatum* may be sensitive to changes in the carbon dioxide to oxygen ratio rather than simply to elevated CO_2_ (Bagby and Chisholm, 2015), due to an unrecognized importance of photorespiration. More research is needed to determine whether photorespiration may play an important role in dinoflagellate metabolism.

**Table 2:** Gene expression of putative CCM and photorespiration genes in six eukaryotic phytoplankton.

	***A. monilatum***	***P. minimum***	***G. oceanica***	***C. polylepis***	***C. affinis***	***H. akashiwo***
**BIOPHYSICAL**						
SLC4	4	2	6	5 (1, 0)	3 (2,0)	3 (2, 0)
Bestrophin	86 (0, 1)	49 (0, 2)	39 (4, 4)	53 (3, 2)	16 (1, 3)	6 (5, 0)
Carbonic Anhydrase α	19	7	2	–	4 (1, 2)	5 (4, 0)
Carbonic Anhydrase β	8	–	–	2 (2, 0)	–	8 (4, 0)
Carbonic Anhydrase δ	5	5	2 (0, 1)	4 (1, 0)	–	–
**C4**						
Pyruvate Kinase (PK)	29 (1, 0)	22 (1, 0)	4 (0, 1)	8 (2, 0)	5 (0, 1)	3 (3, 0)
Phosphoenolpyruvate Carboxylase (PEPC)	1	1	1	1	2	–
Phosphoenolpyruvate Carboxykinase (PEPCK)	8	9	1 (0, 1)	1 (0, 1)	1 (1, 0)	7 (0, 6)
Malate Dehydrogenase (MDH)	24 (1, 0)	22 (0, 1)	4 (0, 1)	2	5	3 (1, 2)
Oxoglutarate/Malate Transporter (OMT)	86 (0, 1)	61 (1, 2)	72 (0, 2)	48 (7, 5)	34 (2, 1)	59 (14, 16)
NAD Malic Enzyme (decarboxylating) (ME)	3	1	2	3	1	3 (0, 1)
Pyruvate orthophosphate dikinase (PPDK)	11	7	–	–	1	–
Pyruvate Carboxylase (PYC)	18	14 (0, 1)	15 (1, 2)	10 (2, 0)	10 (2, 2)	14 (8, 3)
**PHOTORESPIRATION**						
2-Phosphoglycolate Phosphatase (PGP)	12	6	9 (1, 0)	11 (2, 0)	5 (1, 0)	9 (1, 0)
**Mitochondrial pathway**						
Glycolate Oxidase (GOX2)	9 (0, 1)	12 (1, 0)	3	1 (1, 0)	2 (0, 1)	4 (2, 0)
Serine-pyruvate aminotransferase (SPT)	5	3	4	3	1	2 (0, 1)
Alanine Aminotransfirase (ALAT_GGAT)	17	15 (0, 1)	14 (0, 1)	11 (2, 2)	9 (0, 2)	13 (7, 4)
Glycine decarboxylase (GDCT)	10 (0, 1)	8	4	1 (1, 0)	2 (1, 1)	1
Serine Hydroxymethyl transferase (SHMT)	14	20	4	4	3	4 (2, 0)
Hydroxypyruvate Reductase (HR)	30 (1, 0)	18 (0, 1)	10	9 (3, 0)	4	6 (4, 0)
Glycerate Kinase (GK)	1	–	–	–	1	1 (1, 0)
**Peroxisomal pathway**						
Glycolate oxidase (GOX1)	13 (0, 2)	12 (1, 0)	2	1 (1, 0)	1	4 (2, 0)
Malate Synthase (MS)	2	1	–	1 (0, 1)	1 (1, 0)	–
Isocitrate lyase (ICL)	31 (0, 3)	13	4 (0, 1)	3 (0, 1)	3 (1, 0)	4 (0, 3)
**Tartronate semialdehyde pathway**						
Glyoxylate Carboligase (GCL)	13 (1, 0)	4	4	3 (1, 0)	1	1
Tartronate semialdehyde reductase (TSR)	20 (1, 0)	6	7	7 (1, 1)	2	6 (0, 3)

*Expressed homologous contig copy number and in parentheses the number of genes exceeding the expression threshold (log_2_FC >1) and below the threshold (log_2_FC < −1) under elevated CO_2_ respectively. “–“ indicates that no homologous contigs exceeded the minimum count threshold (log_2_CPM > −16)*.

The calcifying haptophyte, *G. oceanica*, displayed no significant difference in CCM or photorespiration gene expression distribution with CO_2_ (Figure 5C). This differed markedly from the non-calcifying haptophyte, *C. polylepis* (Figure 5D), which had significantly different distributions of CCM and photorespiration gene sets with a higher median expression of 25–40%, contrary to expectations of a decreased need for CCM and photorespiration genes under elevated CO_2_. In previous studies with coccolithophores, changes in CCM gene expression and growth rates were only stimulated at much higher CO_2_ levels (Rickaby et al., 2010; Lohbeck et al., 2014). In another study, only a few of the many putative CCM genes were down-regulated in coccolithophores under similar CO_2_ concentrations (Rokitta et al., 2012). Thus, it is possible that coccolithophores may have a threshold response to changing CO_2_ that is only triggered by much larger changes in CO_2_ or that the CCM is controlled by a smaller subset of genes, whose expression is obscured by examining the distribution of all putative CCMs. We speculate that the process of calcification could also uncouple coccolithophores from the impacts of changing CO_2_ by maintaining the need for an energy consuming bicarbonate pump under a wider range of CO_2_ concentrations. This hypothesis is supported by the lack of biologically significant change in the six SLC4 bicarbonate transporter copies for *G. oceanica* (Table 2), but warrants further study.

The diatom, *C. affinis*, significantly shifted the expression of biophysical CCM genes, with the median decreasing by 35% (Figure 5E). The decrease in *C. affinis* biophysical CCM genes with elevated CO_2_ agrees with previous work on the diatom, *T. pseudonana* (Hennon et al., 2014). It may be that the energy saved by decreased expression of biophysical CCM genes (Figure 5, Table 2) allowed for the increase in nitrogen assimilation per cell by *C. affinis* (Table 1), similar to observations of the effects of energy flux on diatom nitrate assimilation (Parker and Armbrust, 2005) and dinoflagellate nitrate assimilation (Eberlein et al., 2016). *H. akashiw*o showed the greatest relative changes in CCM, photorespiration and C4 CCM genes, with a greater than 4-fold increase in median biophysical CCM and a 42% increase in photorespiration, coupled to a 20% decrease in the median C4 CCM gene set expression (Figure 5F). Both CCM gene sets were significantly different in expression distribution when compared to the total gene set. The increase in expression of putative biophysical CCM and photorespiration genes for both *H. akashiwo* and *C. polylepis* is surprising, however it is important to note that the genes in these categories may have multiple functions including acid-base balance of the cell and functions in other metabolic pathways. This re-shuffling of *H. akashiwo* gene expression away from putative C4 genes may have the net impact of increasing energy equivalents for growth and carbon fixation, as evidenced by significantly increased growth rate (Table 1).

Few CCM and photorespiration genes were found to share expression patterns even among the more closely related species (Table 2). All phytoplankton differentially expressed at least one copy of pyruvate kinase (PK), an enzyme central to many metabolic processes including C4 metabolism. There were also changes in phosphoenolpyruvate carboxykinase (PEPCK) and pyruvate carboxylase (PYC) gene expression that exceeded our thresholds (Table 2), however the direction of change was not consistent within a species or among species within the same phyla. These results provide little support for a coherent change in C4 CCM in response to elevated CO_2_ in these phytoplankton species. Within the photorespiration pathway, at least one copy of the gene encoding the enzyme that catalyzes the first step of photorespiration, 2-phosphoglycolate phosphatase (PGP), had increased expression in all four species with form 1D Rubisco (Table 2). This is counter to expectation, as rising CO_2_ should decrease the probability of oxygen being fixed in place of carbon and therefore the need for expressing photorespiration pathways. Other photorespiration genes did have decreased expression including alanine amino transferase (ALAT_GGAT) in the mitochondrial pathway and isocitrate lyase (ICL) in the peroxisomal pathway (Table 2). These gene expression patterns reveal a mixed metabolic pathway response to CO_2_ even within a single species that does not fit the simple picture of how phytoplankton are expected to respond to elevated CO_2_.

Several putative CCM genes displayed a shared response across species but are poorly characterized (Table 2). For example, a family of gated ion channels (bestrophins), closely associated with the expression of other CCM genes (Hennon et al., 2015), met our threshold for differential expression in all six species examined in this study in response to CO_2_ (Table 2). Another gene that was widely shared and regulated in response to CO_2_ was the oxoglutarate/malate transporter (OMT) (Table 2). This gene is also poorly characterized, yet is thought to be essential for C4 metabolic processes, as all eukaryotic phytoplankton have many organelle compartments between the site of C4 fixation and decarboxylation (Kroth et al., 2008). These examples highlight the need for further research on the transporters and ion channels that may serve vital functions in shuttling metabolites between intracellular compartments. These results also suggest that these genes may be more highly-conserved between taxa than other CCM genes.

### Section 3: survey of CCM and photorespiration gene complement of four eukaryotic phytoplankton phyla

**Figure 6:**
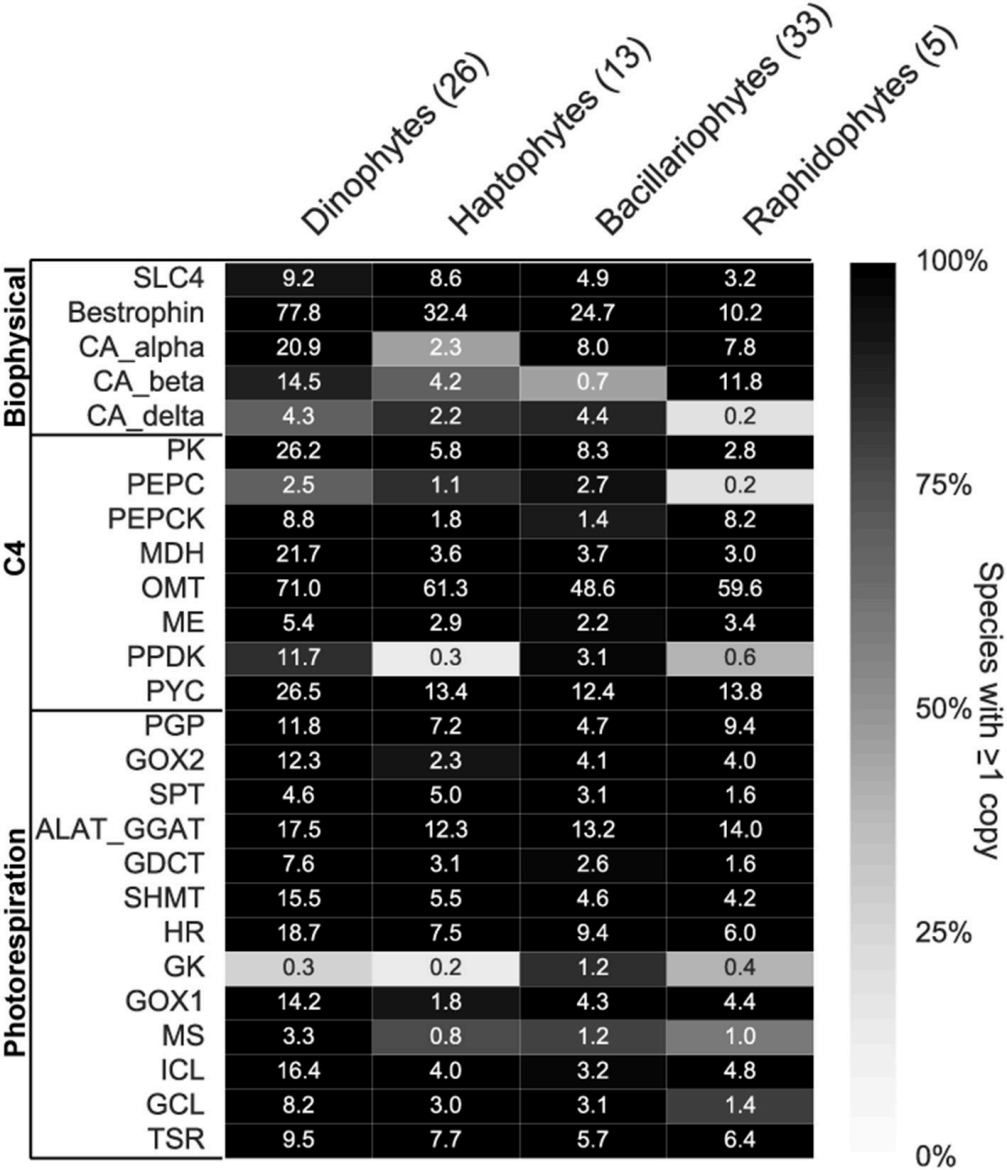
Carbon concentrating mechanism (CCM) and photorespiration expressed gene complement in four phytoplankton phyla. Custom HMM searches for Biophysical (BP) and C4 CCM (C4) and photorespiration (PR) genes within consensus contigs or gene models of each phyla (Supplementary Tables 3, 4). Numbers in parentheses next to phyla name indicates total number of species within each group. Shading indicates percentage of species with at least one copy within each phyla and the numbers indicate mean copy number of each gene. Gene abbreviations are as follows; SLC4, SLC4 bicarbonate transporter; CA, carbonic anhydrase; PK, pyruvate kinase; PEPC, phosphoenolpyruvate carboxylase; PEPCK, phosphoenolpyruvate carboxykinase; MDH, malate dehydrogenase; OMT, oxoglutarate/malate transporter; ME, malic enzyme; PPDK, pyruvate orthophosphate dikinase; PYC, pyruvate carboxylase; PGP, phosphoglycolate phosphatase; GOX, glycolate oxidase; SPT, serine pyruvate aminotransferase; ALAT-GGAT, alanine aminotransferase; GDCT, glycine decarboxylase T-protein; SHMT, serine hydroxymethyl transferase; HR, hydroxypyruvate reductase; GK, glycerate kinase; MS, malate synthase; ICL, isocitrate lyase; GCL, glycolate carboligase; TSR, tartronate semialdehyde reductase.

To assess whether the changes in CCM and photorespiration genes within the six phytoplankton species examined were representative of other phytoplankton within the same phyla, we surveyed genes in these pathways across the consensus assemblies of related species in the MMETSP data set and available genomes (Figure 6, Supplementary Tables 3, 4). A total of 77 species belonging to four phyla were examined (Figure 6). At least one complete photorespiration pathway was expressed in all 77 species (Supplementary Table 3), emphasizing the importance of this pathway for phytoplankton with both forms of Rubisco. Genes for biophysical CCMs were expressed in all species, although the gene type and copy number varied between and within each group (Figure 6). The better-studied CCM gene families like carbonic anhydrases (CA) varied in the type expressed and copy number between phyla (Figure 6). Beta-CAs were prevalent in raphidophytes and dinoflagellates, while delta-CAs were most prevalent in diatoms and haptophytes (Figure 6). Even within diatoms, there was large variation in expression of CA types, with many lacking expression of a beta-CA and expressing only alpha and delta (Supplementary Table 3). No CA family was universally expressed by all groups, suggesting high diversity in the genes used for biophysical CCM, and supporting convergent evolution of some biophysical CCM genes.

Seventy-five out of 77 species expressed a SLC4 bicarbonate transporter gene. The SLC4 transporter has been shown in a diatom to transport bicarbonate into the cell (Nakajima et al., 2013) and is thought to be an integral part of the phytoplankton biophysical CCM. A putative ion channel (bestrophin), identified as a potentially conserved biophysical CCM gene (Hennon et al., 2015, this study), was present in high copy numbers in all 77 species examined (Figure 6). The bestrophin gene family is much less well-studied than SLC4 bicarbonate transporters, yet may serve vital functions for the biophysical CCM (Hennon et al., 2015). Bestrophin genes were likely missed by our orthologous group analysis (Figure 4) because of the abundant paralogous gene copies of bestrophins within each organism (Figure 6, Supplementary Table 3) making it difficult to classify into a single orthologous group. Because bestrophin genes have only been studied in the context of human eye diseases (Yang et al., 2014), their role in phytoplankton CCM remains uncertain.

Putative C4 pathway genes also varied in gene copy and type (Figure 6), with 48 out of 77 species expressing a full complement of C4 genes (Kroth et al., 2008). Similar to the bestrophin gene, the putative OMT transporter for C4 CCMs was universally conserved across the dataset and expressed in high copy numbers (~60 per species, Figure 6). The gene typically considered rate-limiting for the C4 pathway, pyruvate orthophosphate dikinase (PPDK), was not detected in 85% of haptophytes and 60% of raphidophytes (Supplementary Table 3). These patterns indicate that C4 may not be a widespread CCM strategy in haptophytes and raphidophytes, or perhaps that it is expressed at such low levels that it cannot be resolved in the available transcriptomes. The results from the transcriptome survey warrant caution in attributing changes in C4 genes to a change in CCM, particularly given that the localization of these genes is unknown, yet crucial to the functioning of a C4 CCM (Kroth et al., 2008).

## Conclusions

With the recent increase in availability of whole transcriptomes for eukaryotic phytoplankton species (Keeling et al., 2014), it is now possible to ask whether expressed gene complements can inform us about the potential for physiological responses to rising CO_2_. This study focused on characterizing the physiological and gene expression changes of six ecologically important phytoplankton species to elevated CO_2_ in the context of the expressed gene complements from the four phyla they represent. Although some similarities were observed in growth rate and C:N ratio related to phylogeny, changes in gene expression, even those that underpin CCM activity and photorespiration, were largely unique to each species. Many phytoplankton express CCM and photorespiration genes, but the lack of consistent CO_2_ response in the four phyla studied here suggests that responses to CO_2_ perturbations are more diverse than their physiological responses would indicate. As such, extrapolation of detailed genetic mechanisms from a few well-studied phytoplankton to other species, even within the same phylum, may be problematic with regards to CCM and photorespiration pathways. This study demonstrates the value of broad taxonomic comparisons in gene expression studies. Several broadly-defined gene groups or families were highlighted that responded similarly to changing CO_2_ across diverse taxa. Though the functions of those highlighted gene groups, such as bestrophins and oxoglutarate/malate transporters, are poorly understood, identifying such broadly-conserved responses may be required to gauge the response of natural phytoplankton communities to CO_2_ perturbations. The cross-species comparison also revealed divergence in gene complement and regulation between species, providing insight into the physiological and genetic flexibility with respect to changing CO_2_ inherent within phytoplankton communities. Understanding the diversity in genetic complement and gene expression response to elevated CO_2_ can improve predictions of the relative success of phytoplankton taxa in future ecosystems, their impacts on biogeochemical cycles, and harmful algal bloom frequency.

## Author contributions

AJ and SD designed the experiments, AJ and SH performed the experiments, GH and MH analyzed the data, GH wrote the manuscript with input from all authors.

### Conflict of interest statement

The authors declare that the research was conducted in the absence of any commercial or financial relationships that could be construed as a potential conflict of interest.
